# Prize Essays Texas State Medical Association—Circular

**Published:** 1886-03

**Authors:** 


### CIRCULAR.

### $100 GOLD PRIZE FOR BEST ESSAY, BY TEXAS STATE MEDICAL ASSOCIATION.

CLEBURNE, TEXAS, February 22, 1886.

My Dear Doctor:

AS chairman of the Committee on "Prize Essays," I would call your attention to the resolution, adopted at the last session of the State Medical Association, offering one hundred dollars, for "the best original Essay or paper on any medical subject," to be decided at the next annual meeting. We trust you will take a deep interest in this effort of the Association to arouse greater interest in the meetings and favor the Association with an article.

The Essay should be headed by a motto in the English or German language, without the author's signature, and enclosed to the chairman of the committee, with motto endorsed. Then the author's name with his motto, should be enclosed in a separate envelope, which is not to be opened, if paper is rejected. The essay should be in the hands of the committee by the 20th of April, 1886.

Yours Respectfully,

JAS. D. OSBORN, Chair'n,
D. R. WALLACE,
S. D. THURSTON,
H. K. LEAKE,
E. L. THOMPSON,
Committee.

